# A Case of Fibrous Cancer of the Stomach

**Published:** 1869-01-01

**Authors:** R. G. Bogue

**Affiliations:** Surgeon to Cook County Hospital, Chicago


### A CASE OF FIBROUS CANCER OF THE STOMACH.

By R. G. Bogue, M.D., Surgeon to Cook County Hospital, Chicago.

W. S. Bogue, aged sixty-nine, has always been a healthy man, except about twenty-four years ago, when he was sick of inflammatory rheumatism for about six months; since which time he has had hardly a sick day, until July, 1867, when he began to complain of some slight dyspeptic symptoms; noticing that some accustomed article of food did not relish as well as usual, or positively disagreed with him — but seldom was there any pain or distress after eating. A good deal of gas was eructated, no pain nor tenderness in the epigastrium, bowels regular, no vomiting, no nausea, emaciation gradual and persistent, also gradual loss of vigor. This condition continued for seven or eight months without abatement, and without any particular suffering. Some article of food would be relished for a time, then be loathed, giving place to some other, to be in its turn treated the same way. During all of this time there was constant loss of flesh and strength. In March or April of the present year (1868), there began to be rather more distress in the region of the stomach, with a little tenderness on pressure, no definite tumor, but an induration could be distinguished, supposed to be of the stomach or pancreas — there now began to be spat up about half a pint of clear mucus daily, which seemed to come from the œsophegus, as there was no cough whatever, and no condition of the fauces to produce it. Occasionally some article of food would be ejected from the stomach soon after being taken. There was always more distress when lying down, particularly after having been asleep for a time, which seemed to be from an accumulation of gas, for it would be relieved by moving about, which would cause the eructation of quite a quantity of gas; bowels inclined to be constipated.

From this time forward the history was briefly an increase of all the symptoms, making it a case of malignant disease of the stomach, more pain, tenderness, increased tumefaction, occasional vomiting, total loss of appetite, and more rapid emaciation and loss of strength. During the latter part of June, and early part of July, he was quite unable to take any thing into the stomach, except a little wine, and could take at a time not more than two or three teaspoonfuls of that — any thing except the wine caused a good deal of distress, and frequently vomiting.

About July 4th, any attempt to introduce nourishment into the stomach was abandoned; and extract of beef, by injections, substituted — a table-spoonful of the extract dissolved in about four ounces of warm water, was introduced into the rectum four or five times a day, which was, after a few days, increased to two table-spoonfuls. This constituted all the nourishment used until death — for nearly two months, except during the last week, when an egg, beaten thoroughly, with a little syrup and wine, was used two or three times a day in addition to the former.

When this mode of nourishing was begun, he had been confined to the bed for a week or more—from this he seemed to get more nourishment, and was relieved from pain, and was able to be up about the house and yard, and even come to the city from Hyde Park, some four or five times in the course of two or three weeks; yet the emaciation continued, and the temporary increase of vigor soon began to diminish, and for the last two weeks preceding death he was confined to the bed, unable to speak above a whisper; and during the last four days only semi-conscious, seeming to suffer but little, dying very quietly on the morning of the 29th of August, 1868. About a week before death there was an attack of hæmorrhage, some two or three ounces of blood being spat up without distress or effort, this was the only hæmorrhage that occurred during the course of the disease.

During the early part of the disease, and in fact for the first six or seven months, the diagnosis was obscure; rendered so by the absence of the more prominent symptoms of malignant disease of this organ, viz., vomiting, pain, and detection of a tumor; but this condition was suspected from the persistence of the dyspeptic symptoms, with the constant emaciation and loss of vigor.

During this time some of the ordinary remedies for dyspepsia were used, such as *Bismuth*, and *Rhei*, *Sodae* and *Rhei*, *Rennet*, wine, and a few other similar ones, together with bathing and friction over the abdomen, allowing the patient to select for food whatever he seemed to relish.

The remedies, of course, did no good, not even temporarily. After it became quite evident what the malady was, the only thing used was *Elixir Opii*, ten to twenty drops two or three times a day, to relieve distress, and to check the secretion of mucus, which it did; a small blister was applied over the epigastrium, at two different times, which relieved the pain somewhat, temporarily.

*Post-mortem* examination, twelve hours after death. Body extremely emaciated, rigor mortis moderate. Contents of thorax, lungs, and pleuræ perfectly healthy; heart and pericardium normal; aorta, from its very commencement at the heart, throughout its whole extent, in this cavity and the abdominal, to very near its bifurcation, involved extensively by œtheomatous degeneration, but this was not noticed in any of the other vessels. Abdominal cavity and liver normal; spleen, in appearance, healthy, but very small, weighing only one and three-fourth ($1\frac{3}{4}$) ounces; intestines normal; kidneys normal; bladder, etc., normal; pancreas normal. Stomach extremely contracted, with its walls excessively thickened, being three-eighths ($\frac{3}{8}$) of an inch thick where it was opened, which was along the anterior wall, from cardiac orifice to the pylorus. The largest or thickest part was that of the left end of the organ, or that corresponding to the great cul de sac. The whole wall was hard, cutting very toughly, the peritoneal, muscular, and mucous coats seemed blended into one mass, there being no trace of mucous membrane as such, except a small patch near the pylorus; it was not destroyed by ulceration, but seemed to be obliterated or replaced by the infiltration; there was no ulcerated surface. The disease was confined to the walls of the stomach, mostly stopping abruptly at the æsophagus, and also at the duodenum; the pylorus being large, and hanging in the duodenum like an os uteri in the vagina. The duodenum and æsophagus were perfectly healthy. The gastro-colic omentum was somewhat infiltrated and contracted, and the transverse colon adherent to the mass, but the intestinal wall was not diseased. A few lymphatic glands in this part of the omentum were slightly enlarged, but none others were noticed to be infiltrated.

The peritoneal covering of the diaphragm throughout its whole extent, was thickened to the extent of half a line or more, with this same infiltration, feeling hard, and in places being a little corrugated, these places resembling cicatrices. It was contracted, for the partition was more tender than natural, and not arched upward as much as usual.

No other portion of the peritoneum lining the cavity, or covering the intestines, was in this condition, but was, to all appearance, healthy.

The stomach measured, along the small curvature, three and a half (3½) inches; along the cavity, from the cardiac orifice to the pylorice, was four and a half (4½) inches; and around the great curvature it measured eight and a half inches. The cavity was so much contracted that it would not hold more than an ounce and a half of fluid. The whole wall was so thick and unyielding that it could not be distended. The involvment of the entire stomach in cancerous disease is rare, and for it to be infiltrated and contracted throughout, and being confined almost wholly to that organ, as in this case, must be exceedingly rare. The absence of any tumor led to obscurity in the diagnosis, neither was there any of the sallow complexion, which is so generally looked upon as evidence of cancerous disease. But its malignant character was inferred from the persistence of the symptoms, and the gradual but constant emaciation. And from the increasing inability to take food it seemed that the stomach must be the organ implicated.

There is one thing that I would call particular attention to in this case: which is the length of time the patient was kept alive by nutritive enema. This was for nearly two months, or fifty-five days. After this was commenced, the strength of the patient increased for some weeks, and during all of this time there was no sensation of hunger; yet there was constant emaciation, and death seemed to be the result of absolute starvation.

The inference I draw from this is, that life may be prolonged for a considerable time, by this mode of introducing nourishment, but the body is not nourished. In other words, there seems to be enough nutriment absorbed to continue life, but there is no surplus appropriated. The fact that a person may be sustained for so long a time in this way, may be a valuable hint in cases of gastric affection, where it is essential to leave this organ at rest for some time, also in operation upon the æsophagus or mouth.

Under the microscope this seems to be composed of fibrous tissue mostly, with a few irregular cells, having the appearance of being pinched or contracted by the fibrous material surrounding them.

